# Ultrasonic-assisted production of okara protein isolate amyloid fibrils from plant-based by-products: Structural and morphological characteristics

**DOI:** 10.1016/j.ultsonch.2026.107857

**Published:** 2026-04-16

**Authors:** Shuangshuang Wang, Haokun Zhang, Huilin Lv, Zhenzhu Li, Shanshan Tie, Fang Zhao, Peifeng Li

**Affiliations:** a College of Food and Bioengineering, Zhengzhou University of Light Industry, Zhengzhou 450001, China; b Key Laboratory of Cold Chain Food Processing and Safety Control, Ministry of Education, Zhengzhou University of Light Industry, Zhengzhou 450001, China; c Institute of Life and Health, Zhengzhou University of Light Industry, Zhengzhou 450001, China; d National & Local Joint Engineering Research Center of Cereal-Based Foods (Henan), Zhengzhou 450001, China; e College of Food and Bioengineering, Henan Engineering Research Center of Food Microbiology, Henan University of Science and Technology, Luoyang 471023, China; f Henan Institute of Product Quality Inspection Technology, Zhengzhou 450001, China

**Keywords:** Okara protein isolate, Plant-based by-products, Amyloid fibrils, Ultrasonic pretreatment, Structural characteristics

## Abstract

The amyloid fibrilliation technology of plant proteins points to the development of new functional foods using plant processing by-products. Okara protein isolate (OPI) is such a sustainable protein resource from soybean processing, with huge output and rich nutrition. This study employed ultrasonic pretreatment to enhance the fibrillation capacity of OPI. Following ultrasonic pretreatment, heating at 90 °C under pH 2 conditions for 9 h yielded amyloid fibrils with high conversion rates, validated through ThT fluorescence, SDS-PAGE, and AFM techniques. UV and FTIR analyses confirmed the formation of amyloid fibril structures rich in characteristic cross-β-sheets. Furthermore, this study revealed that ultrasonic pretreatment may partially unfold proteins and expose hydrophobic regions, subsequently promoting amyloid fibril aggregation during incubation. Conversely, excessive ultrasonic pretreatment may lead to over-unfolding of protein structures, resulting in structural instability that hinders amyloid fibril aggregation. This research not only elucidates the mechanism by which ultrasound regulates amyloid fibril formation but also provides a practical solution for the application of soybean residue protein in the field of food science.

## Graphical abstract

**Figure:**
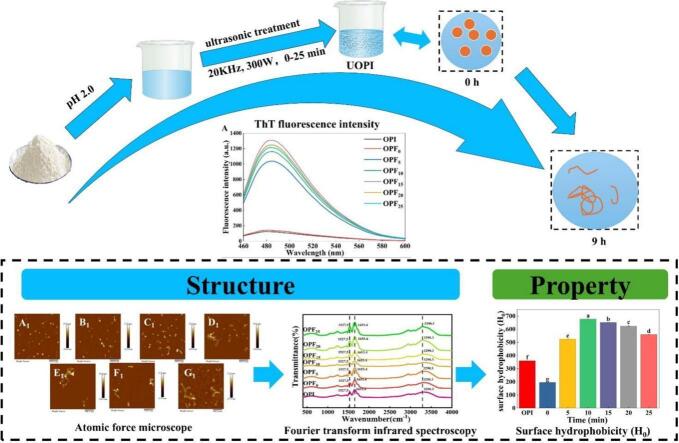

## 1 Introduction

Okara is a by-product generated during the production of soy-based products. Approximately 1.2 kg of wet okara is produced from every kilogram of processed soybeans [1]. The global annual production of okara amounts to 140 million tons, with China contributing 2.8 million tons to this total [2]. Although soy is frequently utilized as an ingredient in animal feed, the presence of specific compounds can impede protein absorption and increase the risk of digestive disturbances, such as diarrhea and indigestion [3]. Okara is both economically viable and nutritionally beneficial, exerting positive effects on physiological metabolism. Nevertheless, its application is constrained by several factors, including a coarse texture, suboptimal palatability, elevated moisture and protein levels, and a pronounced susceptibility to spoilage [4]. Additionally, the action of lipoxygenase in soybeans on lipids produces volatile aldehydes, ketones, and alcohols, which contribute to an undesirable beany flavor [5]. These factors pose significant challenges to the development and utilization of okara.

Amyloid fibrils, also known as protein fibrils, represent a unique aggregation behavior exhibited by certain proteins and peptides [6]. Under specific conditions, proteins or peptides can self-assemble into fibrous structures, which are referred to as amyloid fibrils. This self-assembly process occurs when native proteins are subjected to specific environmental conditions, such as appropriate temperature, pH, ionic strength, or enzymatic treatment [[7], [8]]. The formation of these protein amyloid fibrils needs two conditions [9], which are a sufficiently high protein concentration and a heating temperature that must exceed the protein's denaturation temperature. Currently, fibril assembly is commonly induced by acid–heat treatment, typically performed at pH 2.0 under prolonged heating. Moreover, research has demonstrated that the majority of food-grade globular proteins are capable of self-assembling into fibrillar structures via hydrophobic interactions, van der Waals forces, and electrostatic interactions. The interplay and synergy of these intermolecular and intramolecular interactions are fundamental to the fibrillation process [10]. The exposure of internal hydrophobic groups within the protein can also increase the opportunity for intermolecular contact, thereby promoting fibrillar self-assembly [11]. Moreover, due to the unique structure of amyloid fibers, they have garnered significant attention in the field of food science. Applications include thickening agents [12], emulsifiers and foam stabilizers [13], carriers for bioactive compounds [14], and food packaging [15]. Further research and development can fully unlock their immense potential in the food sector [16].

Ultrasonic technology, a type of mechanical wave distinguished by its strong penetration capabilities, precise directivity, and adjustable frequency, presents several advantages, including operational simplicity, high efficiency, cost-effectiveness, non-toxicity, environmental sustainability, and enhanced safety [17]. The cavitation, mechanical, and thermal effects generated by ultrasound can enhance the processing quality of raw materials and increase production efficiency [18]. Studies have shown that ultrasonic pretreatment can unfold protein structures, leading to the exposure of more binding sites [19]. During the formation of protein amyloid fibrils, the cavitation effect and subsequent bubble collapse generate powerful shear forces. These forces accelerate the spontaneous nucleation of monomeric proteins and cause fragmentation of existing fibrils, ultimately yielding short and uniform amyloid-like fibrils [20]. Based on the aforementioned evidence, we hypothesized that the combined ultrasonication-assisted and acid-heat treatment would synergistically promote the fibrillation of OPI more effectively than either treatment alone.

The aim of this study was to examine the formation of amyloid-like fibrils from OPI utilizing ultrasonication-assisted acid-heat treatment. This methodology integrates the protein unfolding effects induced by ultrasound with traditional acid-heat induction to facilitate the rearrangement and ordered self-assembly of OPI molecules into fibrillar structures. The study systematically evaluated the impact of varying durations of ultrasonic pretreatment on the fibrillation process, as well as the resulting structural and physicochemical properties of the fibrils. This was achieved through the application of atomic force microscopy (AFM), polyacrylamide gel electrophoresis (SDS-PAGE), spectroscopy, and chemical analyses. By focusing on okara, a protein-rich yet underutilized by-product, this research provides a comprehensive understanding of how ultrasonic pretreatment alters the fibrillation pathway and affects the functional performance of the resultant fibrillar networks. The findings propose an innovative strategy for the high-value utilization of okara through protein fibrillation, facilitated by the synergistic application of physical and chemical processing techniques.

## 2 Materials and methods

### 2.1 Materials

Okara protein isolate powder was purchased from Shaanxi Herbasea Biotechnology Co., Ltd. (Xi’an, China). BeyoBlue™ Plus Coomassie Brilliant Blue Ultra-Fast Staining Solution was obtained from Beyotime Biotechnology (Shanghai, China). 4 × protein loading buffer (containing DTT) and Tri-color pre-stained protein marker (10–180 kDa) were sourced from Solarbio Science & Technology Co., Ltd. (Beijing, China). Thioflavin T (ThT) and 8-anilino-1-naphthalene sulfonic acid (ANS) were acquired from Yuanye Bio-Technology Co., Ltd. (Shanghai, China). All other chemicals used in this study were of analytical grade.

### 2.2 Preparation of okara protein isolate amyloid-like fibrils (OPF)

OPF was extracted according to the previous methods [[21], [22]] with slight modifications, and all samples underwent the same subsequent steps of overnight storage, centrifugation, and heat treatment. The OPI sample was mixed with deionized water at a ratio of 3% (w/v). The pH of the solution was then adjusted to 2.0 using 1.0 M HCl, followed by magnetic stirring at 25 °C for 2 h. Subsequently, the mixture was subjected to ultrasonic pretreatment using an ultrasonic cell disruptor (SN-PS250, Guangdong Jepon Ultrasonic Industrial Co., Ltd., Guangdong, China). The parameters were set at a frequency of 20 kHz with a pulse mode of 2 s on and 2 s off and a power of 300 W. During sonication, the sample was placed in an ice bath to maintain a low temperature. The ultrasonic pretreatments were applied for different time intervals (0, 5, 10, 15, 20, and 25 min). The resulting samples were labeled as OPF_x_, where 'x' represents the ultrasonic time. Notably, prior to sonication, all protein solutions were derived from the same concentration of stock solution and aliquoted into separate centrifuge tubes to ensure consistent initial protein concentrations across all samples. Following ultrasonic pretreatment, all protein solutions were stored at 4 °C overnight to ensure complete hydration. Following this, the samples were centrifuged at 8000 × *g* and 4 °C for 30 min. The resulting supernatant was transferred into sealed centrifuge tubes containing a stir bar to ensure uniform heating. The tubes were then heated in a magnetic stirring water bath at 90.0 ± 2.0 °C for 9 h. Immediately after the heat treatment, the samples were cooled in an ice-water bath to room temperature for subsequent analysis.

### 2.3 Characterization of the OPF

#### 2.3.1 Thioflavin T (ThT) fluorescence spectra

Thioflavin T (ThT) is one of the most widely employed standards for the identification of amyloid fibrils [[23], [24]]. In this study, the assay was the previously published study of Yang et al. [25] with slight modifications. The ThT stock solution (0.8 mg/mL) was prepared in phosphate-buffered saline (150 mM, pH 7.2) and stored at 4 °C protected from light. Prior to detection, the stock solution was diluted 50-fold with the same buffer to obtain the working solution. Forty microliters of protein sample were mixed with 1.5 mL of ThT working solution as described, followed by fluorescence measurement. Fluorescence signals were measured using a fluorescence spectrophotometer (F-7100, Hitachi, Tokyo, Japan) with an excitation wavelength of 410 nm. The emission spectrum was scanned over 460–600 nm with a slit width of 10 nm.

#### 2.3.2 Turbidity analysis

Turbidity measurements were conducted according to a previously published method [26] with minor modifications. A 0.1% (w/v) sample solution was prepared using deionized water at pH 2, and the transmittance (T) of the sample solution was measured at a wavelength of 600 nm using a visible spectrophotometer (722 N, Shanghai Jinghua Technology Instrument Co., Ltd., Shanghai, China). Deionized water at pH 2.0 served as the blank control, with turbidity expressed as 100 − T%.

#### 2.3.3 Determination of surface hydrophobicity (H_0_)

The surface hydrophobicity of the samples was determined using 8-anilino-1-naphtha lene-sulfonic acid (ANS) as a fluorescent probe, following a previously published method [27] with some modifications. 8 mM ANS solution was prepared by dissolving ANS powder in phosphate buffer (pH 7.0). The OPF solutions were diluted to various concentrations (0.005%, 0.01%, 0.015%, and 0.02% w/v). Then, 2 mL of each diluted sample was thoroughly mixed with 50 μL of the ANS solution and incubated in the dark for 15 min. The fluorescence intensity was measured using a fluorescence spectrophotometer (F-7100, Hitachi, Tokyo, Japan) with the excitation wavelength set at 390 nm and the emission spectrum recorded from 410 nm to 650 nm. The fluorescence intensity was plotted against the sample protein concentration, and the slope of the resulting linear regression equation was defined as the surface hydrophobicity (H_0_) [28].

#### 2.3.4 Zeta potential and particle size measurements

Particle size was measured by dynamic light scattering using a Nanoparticle Size and Zeta Potential Analyzer (Zetasizer Nano ZS90, Malvern Panalytical, Malvern, U.K.). The sample was diluted to a concentration of 1.0 mg/mL using deionized water at pH 2.0 for the determination of its average particle size and zeta potential [29].

#### 2.3.5 Determination of UV–vis spectra

Based on a previously published method [30] with modifications, all samples were diluted to a concentration of 1.0 mg/mL using deionized water at pH 2.0. Ultraviolet–visible (UV–Vis) scanning was performed from 200 to 400 nm at 25 ℃ using a microplate reader (ZDM-ScanB, ZhuoDi Instrument Equipment Co., Ltd., Shanghai, China). The absorption spectrum within the 210–320 nm range was obtained for analysis.

#### 2.3.6 Fourier transform infrared spectroscopy (FTIR)

Changes in functional groups were detected using an FTIR spectrometer (Vertex 70, Bruker, Germany), following a previously published method [31] as described in the following. The freeze-dried samples were mixed with spectroscopic-grade KBr powder at a ratio of 1:100 (w/w) and finely ground using an agate mortar and pestle. The anhydrous mixture was compressed into transparent pellets using a pellet press. The pellets were then scanned in the FTIR spectrophotometer with a resolution of 4 cm^–1^ and 16 scans over a wavenumber range of 400 to 4000 cm^–1^. The amide I band (1700–1600 cm^–1^) of the infrared spectra was analyzed using PeakFit software (version 4.12, Systat Software, Inc., USA) to determine the secondary structure content of the protein samples. The spectra were subjected to Gaussian fitting to obtain the curve-fitted profiles of the amide I band [32].

#### 2.3.7 Sodium dodecyl sulfate polyacrylamide gel electrophoresis (SDS- PAGE)

SDS-polyacrylamide gel electrophoresis (SDS-PAGE) was performed according to a previously published method [33] with minor modifications. All samples were diluted to 2.0 mg/mL with deionized water (pH 2.0) and centrifuged at 5000 × *g* for 10 min. The resulting supernatant was mixed with a 4 × protein loading buffer (P1015, Solarbio). The mixture was vortexed thoroughly, heated in boiling water for 10 min, and then cooled for subsequent use. Precast gradient gels (4–12%, P41212, Solarbio) were used. A protein molecular weight marker (10–180 kDa) was employed as a standard. Samples (10 μL) and the marker (10 μL) were loaded onto the gel. Electrophoresis was carried out using a Bio-Rad PowerPac Basic power supply (Bio-Rad Laboratories, Shanghai, China) at constant voltages of 200 V and 25 mA until the dye front reached the bottom of the gel. The gel was then stained with Coomassie Brilliant Blue fast staining solution (Beyotime Biotechnology, Shanghai, China) with horizontal shaking for 30 min, followed by destaining with deionized water.

#### 2.3.8 Atomic force microscopy (AFM)

Based on a previously published method [34] with modifications, the amyloid fibril solution was diluted to 0.05 mg/mL using a deionized water solution at pH 2.0. A 10 μL aliquot of the diluted solution was deposited onto a freshly cleaved mica surface and allowed to adsorb for 2 min. The mica sheet was then gently rinsed with 1 mL of deionized water (pH 2.0). After allowing the sample to settle for an additional 2 min, it was rinsed with deionized water (pH 2.0) and dried using compressed air. After drying, the samples were scanned using a Bruker Dimension ICON atomic force microscope (Bruker, Germany) at a scan rate of 3 kHz. The resulting images were captured and saved. Subsequently, the AFM images were analyzed with NanoScope 1.5 software to determine the diameter and length of samples.

### 2.4 Statistical data analysis

All experiments were independently performed in triplicate, and the results are expressed as the mean ± standard deviation. Statistical analysis was performed using SPSS software. Analysis of variance (ANOVA) followed by Duncan's multiple range test was used to determine significant differences (*P* < 0.05) among samples with different ultrasonic times.

## 3 Results and discussion

### 3.1 ThT fluorescence assay

**Fig. 1:**
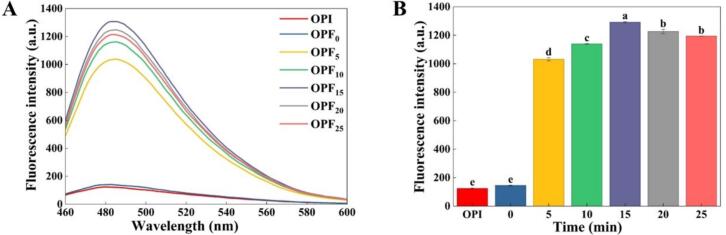
Th T fluorescence spectra of okara amyloid fibrils at different ultrasonic treatment times. (A) Total fluorescence spectra; (B) Fluorescence intensity at 480 nm. Different letters indicate significant differences (*P* < 0.05).

Monomeric proteins can form amyloid fibrils characterized by β-sheet structures through misfolding and side-chain interweaving. ThT, a fluorescent dye that specifically binds to the β-sheet structures within amyloid fibrils, is widely recognized as an effective method for detecting amyloid fibril formation [35]. As shown in Fig. 1A, the ThT fluorescence intensity of OPF was significantly higher than that of OPI, indicating that ultrasonic pretreatment exposed more binding sites in the okara protein, which facilitated the formation of amyloid fibrils rich in β-sheet structures and consequently enhanced ThT binding [22]. Fig. 1B displays the variation in their maximum fluorescence intensities. The fluorescence intensity of OPF exhibited an upward trend during the first 15 min of ultrasonic pretreatment, increasing from an absorbance of 1031.3 ± 4.53 at 5 min to a peak of 1292 ± 7.81 at 15 min. Thereafter, it began to decline, reaching an absorbance of 1194 ± 0.58 at 25 min. A similar phenomenon was also reported by Mantovani et al. [36] under extended ultrasonic pretreatment conditions. This indicates that ultrasonic pretreatment lasting up to 15 min promotes the exposure of binding sites, thereby facilitating amyloid fibril formation. Conversely, ultrasonic pretreatment exceeding 15 min causes excessive unfolding of the protein structure, which is detrimental to the formation of β-sheet structures and consequently reduces ThT binding capacity.

### 3.2 Turbidity

**Fig. 2:**
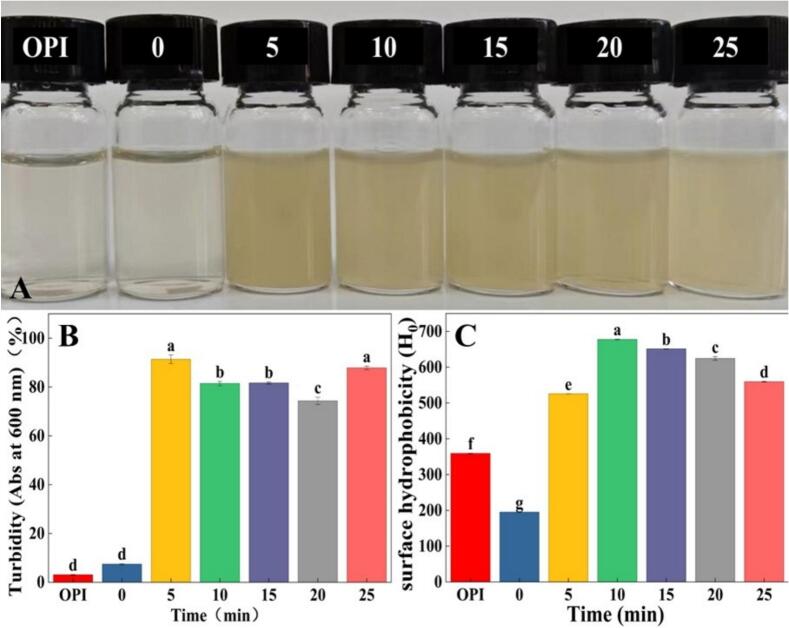
(A) Visual appearance, (B) turbidity, and (C) surface hydrophobicity of OPF subjected to different ultrasonic treatment times (0, 5, 10, 15, 20, 25 min) and untreated OPI. Different letters indicate significant differences (*P* < 0.05).

As clearly shown by the turbidity analysis (Fig. 2A and 2B), significant changes occurred in protein aggregates [37]. The supernatant of untreated OPI at pH 2.0, below the isoelectric point of the proteins, resulting in relative clarity, which is consistent with the findings of Cai et al. [38]. The acid-heat treatment used to prepare OPF only slightly increased the turbidity, aligning with previous studies on chickpea proteins [39]. This may be because OPI self-assembled into a macromolecular polymer, leading to an increase in turbidity [26]. Interestingly, in Fig. 2B, after ultrasonic pretreatment, the turbidity increased significantly. This may be because ultrasonic pretreatment unfolded the native structure of proteins, and these unfolded molecules reassembled into an extremely large number of nanoscale aggregates. Turbidity is related to the total scattering cross-sectional area of all particles in the solution [40]. Although the scattering ability of individual particles was weak, the significant increase in the total number of particles resulted in a substantial increase in both the total scattering area and particle number density, ultimately manifesting as an increase in turbidity. Similar to the trend observed with Rafiei et al. [41]. Concurrently, Fig. 2B demonstrates that extending the initial phase of ultrasonic pretreatment reduces turbidity, whereas excessive processing causes turbidity to rise again. This phenomenon may be attributed to ultrasound-induced structural alterations in OPI. Our results are consistent with the results of the present study [42], which reported that moderate ultrasonication-assisted disruption of hydrophobic regions and exposure of hydrophilic groups, enhance protein solubility. However, excessive treatment induces structural over-disruption, which compromises solubility. These turbidity variations were similar to those found by Rafique et al. [43] in oat protein.

### 3.3 Surface hydrophobicity (H_0_)

ANS can be used as a fluorescent probe to detect hydrophobic groups on protein surfaces [44]. The surface hydrophobicity of OPF formed by acid-heat treatment, as shown in Fig. 2C, was significantly lower than that of OPI. This reduction is attributable to the sequestration of hydrophobic groups during the self-assembly process of amyloid-like fibrils, wherein hydrophobic regions interact and become incorporated into the fibrillar architecture [45]. Similar phenomena have been observed in rice glutelin [46] and ovalbumin [47]. In contrast, ultrasonic pretreatment significantly increased the surface hydrophobicity of OPF compared to OPI, as the intense mechanical effects of ultrasound promote molecular motion and expose previously buried hydrophobic residues [48]. This observation is explained by Huang et al. [37], who demonstrated that the extent of hydrophobic interactions during protein aggregation can be reflected by turbidity measurements. Moreover, the turbidity trend illustrated in Fig. 2B substantiates the hypothesis that variations in turbidity are correlated with the quantity of hydrophobic structures. Specifically, moderate ultrasonic pretreatment facilitates the exposure of hydrophobic groups, whereas excessive treatment results in their disruption. Previous studies have reported similar results for soy protein [49].

### 3.4 Analysis of particle size and zeta potential

**Fig. 3:**
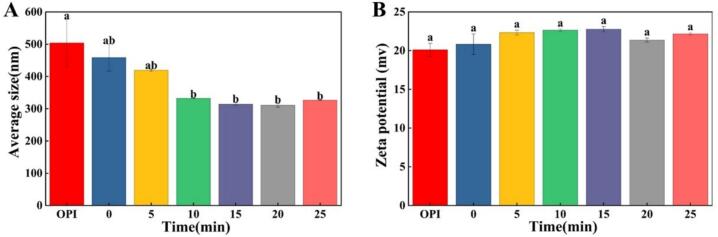
(A) Mean particle size and (B) zeta potential of OPF subjected to different ultrasonic treatment times (0, 5, 10, 15, 20, 25 min) and untreated OPI. Different letters indicate significant differences (*P* < 0.05).

Particle size measurement is widely used to study the aggregation behavior of protein particles and amyloid fibril formation [50]. The analysis of mean particle size and zeta potential can be seen from Fig. 3. As shown in Fig. 3A, the average particle size of OPI decreased from 503.8 nm in the untreated sample to 458.3 nm after acid-heat treatment and further to 310.6 nm following ultrasonic pretreatment, indicating that ultrasonic pretreatment significantly reduced the particle size of OPF. This trend is consistent with our previous findings in chickpea protein [39]. Zeta potential is a critical parameter for characterizing proteins, as it reflects the net surface charge of particles and provides insight into their surface charge state, structural changes, and stability [51]. When the absolute value of the zeta potential surpasses 20 mV, the sample achieves stable dispersion within the solvent. Moreover, a greater absolute value signifies increased electrostatic repulsion among molecules, thereby enhancing the stability of the dispersion [52]. Fig. 3B clearly shows that the absolute zeta potential of OPI was approximately 20 mV, while all OPF samples exhibited values above this threshold. Notably, when ultrasonic pretreatment exceeded 15 min, the absolute zeta potential decreased, suggesting that moderate ultrasonic pretreatment promotes a more stable and uniform dispersion of OPF in solution, whereas excessive treatment may disrupt the protein structure, leading to instability. A similar pattern has been reported in studies on soy protein [50]. ThT fluorescence (Fig. 1B), turbidity (Fig. 2B), and surface hydrophobicity (Fig. 2C) assays indicated that ultrasonication-assisted beyond 15 min disrupted the OPI structure, leading to decreased solution stability and impaired fibril formation. The analysis of particle size and zeta potential provided additional corroboration for this conclusion.

### 3.5 UV spectra

**Fig. 4:**
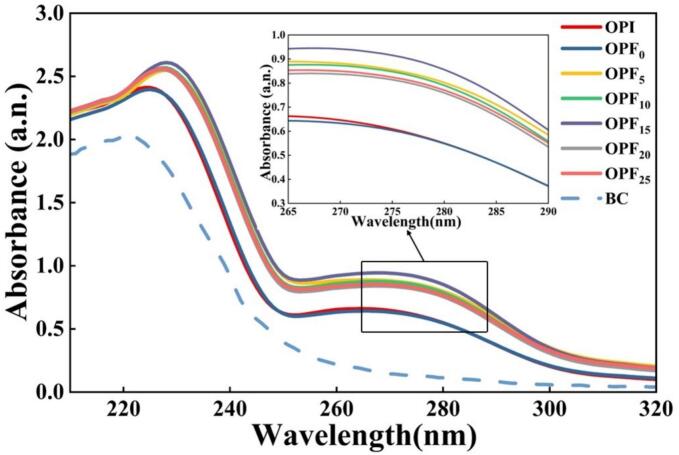
UV absorption spectra of OPF subjected to different ultrasonic treatment times (0, 5, 10, 15, 20, 25 min) and untreated OPI. BC represents the blank control group (pH 2.0 pure water).

The UV absorption spectra of OPI and OPF are shown in Fig. 4. Two characteristic absorption peaks were observed near 230 nm and 275 nm, which are attributed to the peptide bonds (C=O) and aromatic amino acid residues such as tryptophan, tyrosine, and phenylalanine in proteins, respectively [53]. As shown in Fig. 4, OPF generated under acid-heat conditions exhibited increased absorbance around 260 nm, while subsequent spectral changes were similar to those of OPI. This suggests alterations in protein structure leading to increased exposure of tyrosine and tryptophan residues, which may be associated with the formation of amyloid-like fibrils in okara protein [54]. Following ultrasonic pretreatment, the overall absorbance was higher than that of the non-ultrasonic pretreatment group, and a redshift of λmax was observed. These phenomena indicated an increase in the polarity of the protein solution and enhanced conjugation within the protein structure, resulting in a reduction in the energy required for electronic transitions. These changes may facilitate protein fibrillation [55]. Similar phenomena have been reported in studies on faba bean protein [56] and distiller's grain protein [57].

### 3.6 Fourier transform infrared spectroscopy analysis

**Fig. 5:**
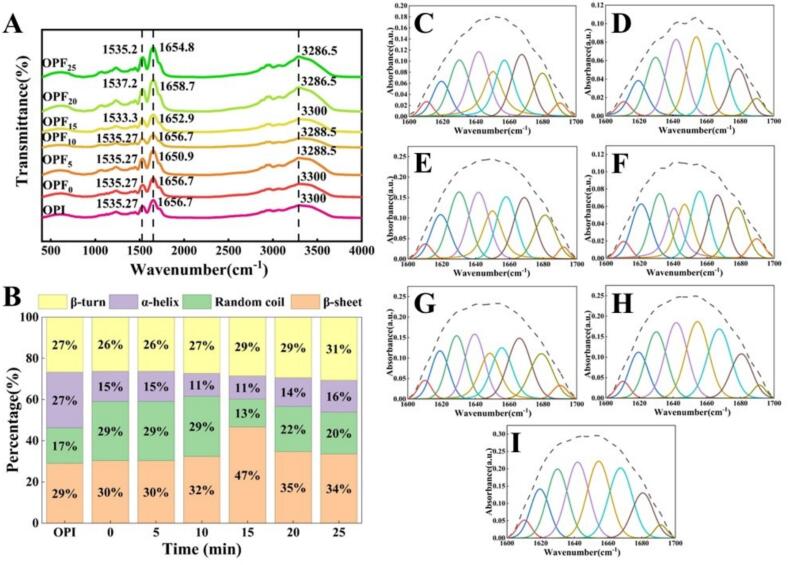
(A) Fourier transform infrared (FTIR) spectra of OPF subjected to different ultrasonic treatment times and untreated OPI. (B) Secondary structure components obtained by deconvolution and fitting of the amide I region in the FTIR spectra. (C-I) Deconvoluted amide I bands of OPF at different heating time points under various ultrasonic treatment durations (OPI and 0, 5, 10, 15, 20, and 25 min).

Fourier transform infrared (FTIR) spectroscopy, which can detect the molecular structure of proteins, was therefore used to analyze the effect of ultrasonic pretreatment on the structure of OPI. Quantitative analysis was achieved using the amide I band in the 1600–1700 cm^–1^ region [58]. The region between 3100 and 3600 cm^–1^ exhibits a broad absorption peak due to the stretching vibrations of O-H and N-H groups involved in intermolecular hydrogen bonding. As shown in Fig. 5A, the peak position shifted slightly from 3300 cm^–1^ to 3286.5 cm^–1^. This shift suggests that hydrogen bond formation exceeded disruption during fibril formation [59], resulting in an extensive hydrogen-bonded network that is essential for stabilizing the β-sheet structure [60]. A similar phenomenon was observed in our previous study on chickpea protein [39], likely attributable to electrostatic interactions and hydrogen bonding among the protein aggregates. According to Oboroceanu et al. [61], a blue shift in the amide I band (1600–1700 cm^–1^) can disrupt hydrogen bonds within the protein structure and promote fibril formation during heating. Spectral changes in the 1480–1590 cm^–1^ range (amide II region) are primarily associated with C=O and N–H bending vibrations (or C–N stretching and N–H bending) [62], and their alterations may be attributed to electrostatic interactions [63]. Analysis of Fig. 5B revealed a significant increase in β-sheet content and a relative decrease in α-helix and random coil structures following ultrasonic pretreatment. This structural transformation arises due to the facilitation of fibril assembly by the formation of β-sheet structures. An increased conversion from α-helix to β-sheet during protein self-assembly results in more significant conformational changes and entails a more extensive reorganization of hydrogen bonds. This phenomenon aligns with the observed peak shift at 3286.5 cm^–1^ [59]. Compared to ultrasonic pretreatment, acid-heat treatment alone had a relatively minor effect on the β-sheet content within the protein amyloid-like fibrils. This observation indicates that ultrasonic pretreatment more effectively promotes the formation of amyloid-like fibrils, a conclusion that coincides with the findings from UV spectral analysis (Fig. 4).

### 3.7 SDS-PAGE analysis

**Fig. 6:**
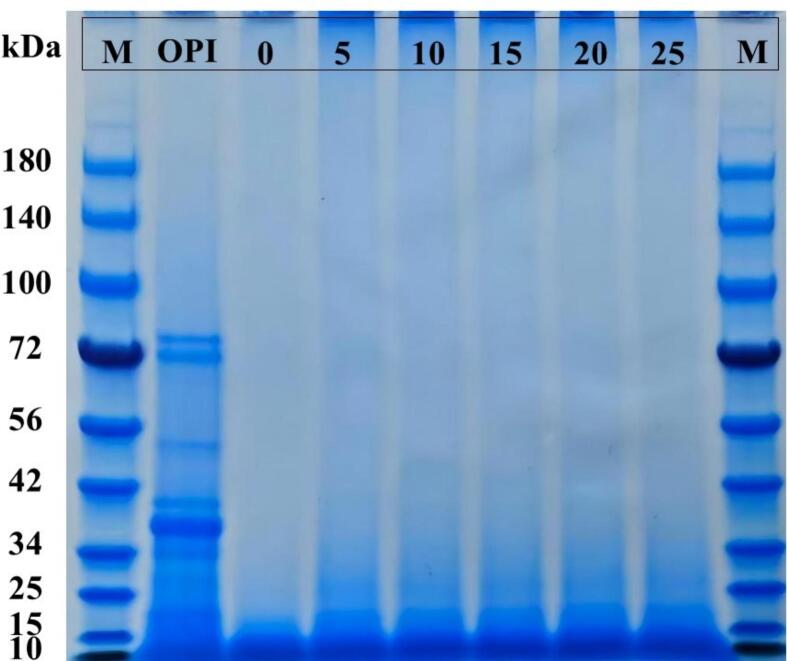
SDS-PAGE analysis of OPF subjected to different ultrasonic treatment times (0, 5, 10, 15, 20, 25 min) and untreated OPI.

SDS-PAGE was used to analyze the changes in OPI subunits during amyloid fibril formation under ultrasonic pretreatment, as shown in Fig. 6. The major protein bands of OPI were observed at approximately 80, 72, 55, 38, and 17 kDa, which is consistent with the band pattern reported by Tang et al. [52] for OPI. The bands at 80, 72, and 55 kDa correspond to the α′, α, and β subunits of β-conglycinin in the 7S globulin fraction, while the 38 kDa band corresponds to the acidic subunit A of the 11S glycinin and the 17 kDa band to the basic subunit B of glycinin [64]. In contrast, OPF generated by acid-heat treatment exhibited a major band near 10 kDa, indicating that the formation of amyloid-like fibrils involves protein denaturation and hydrolysis into peptides, followed by the self-assembly of small peptides into fibrillar structures [65]. When ultrasonic pretreatment was applied, the resulting fibrils showed a predominant band around 15 kDa, suggesting that ultrasound promotes the association of short fibrils into larger aggregates [66]. Notably, large aggregates (>170 kDa) were still detected in samples subjected to ultrasonic pretreatment, suggesting the presence of high-molecular-weight protein polymers that were not completely dissociated under SDS conditions. These aggregates may partly arise from disulfide bond formation, while cavitation effects generated during ultrasonic could further promote protein aggregation. In addition to disulfide linkages, ultrasonic pretreatment may facilitate other covalent interactions [67], including oxidation-mediated crosslinking and aldehyde–amine coupling reactions [68]. Ultrasonication-induced structural unfolding may also expose previously buried reactive amino acid residues, thereby favoring the formation of γ-glutamyl–lysine isopeptide bonds during protein crosslinking [69]. Furthermore, increased exposure of amino groups may enhance Maillard-type glycation reactions, leading to crosslinked structures associated with advanced glycation end products that contribute to the formation of SDS-resistant aggregates [70]. These mechanisms collectively provide plausible explanations for the enhanced aggregation behavior observed after ultrasonic pretreatment and are consistent with previously reported ultrasound-induced cross-linking phenomena [71].

### 3.8 Morphological evaluated via AFM

**Fig. 7:**
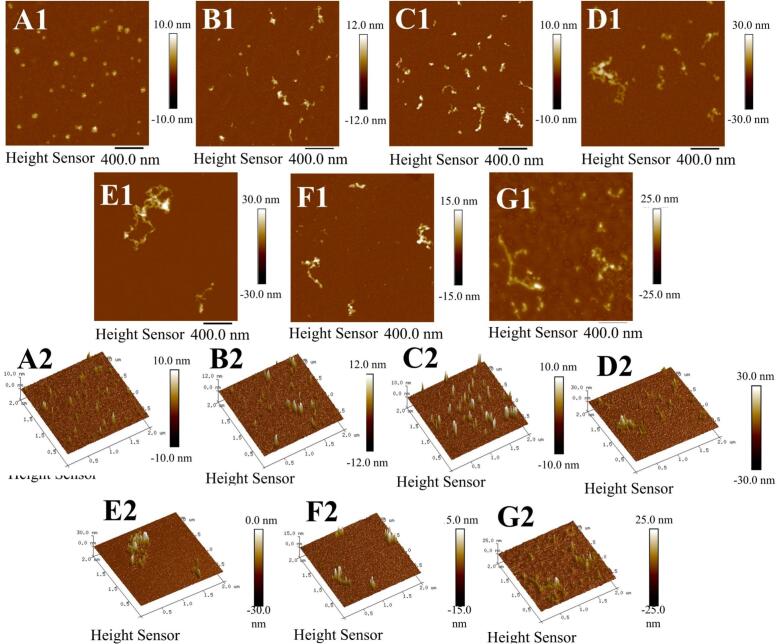
Atomic force microscopy (AFM) images of okara amyloid-like fibrils subjected to different ultrasonic treatment times. Panels 1 and 2 represent the height images and 3D images, respectively. The scale bar in panels A-F is 400 nm. Panel A shows OPI, while panels B-G correspond to OPF treated with ultrasonic treatment for 0, 5, 10, 15, 20, and 25 min, respectively.

The morphological evolution of OPF under different ultrasonic times is shown in Fig. 7A-G. Under heating at pH 2.0 for 9 h, OPI molecules progressively associated and transformed into short fibrillar structures. A similar process of short fibrillar structures has been observed in the study by Xie et al. [72], representing a typical pathway of protein amyloid-like fibrillation. As seen in Fig. 7B-G, ultrasonic pretreatment is beneficial to the association of short fibrillar structures into longer fibrillar structures, which was most evident after 15 min of treatment (Fig. 7E). Nevertheless, excessive ultrasonic pretreatment can induce over-unfolding of the protein structure, thereby modifying the subsequent self-assembly pathway during acid-heat incubation. This alteration leads to the formation of shorter and thicker fibrillar aggregates instead of elongated fibrils. As Table S1 also demonstrates this phenomenon, both fibrillar structures' diameter and length undergo continuous changes with increasing ultrasonic time, reaching a minimum diameter of 3.29 ± 1.09 nm and a maximum length of 31.58 ± 8.45 nm at 15 min. This indicates that the protein has been converted into a fibrous structure, namely OPF. However, ultrasonic pretreatment exceeding 15 min led to a gradual increase in fibrillar structures' diameter and a decrease in length, ultimately forming smaller fragments, a phenomenon similar to that observed by Ji et al. [49]. These observations are consistent with the inferences drawn from the ThT fluorescence analysis discussed above. Combined analysis of UV–visible spectra (Fig. 4) and Fourier transform infrared spectra (Fig. 5A) further confirms that acid-heat conditions promote OPI molecular association into amyloid-like fibrillar fragments. Moderate ultrasonic pretreatment induces partial protein unfolding, exposing hydrophobic regions that facilitate subsequent amyloid fibril formation [50]. However, excessive ultrasonic pretreatment may lead to excessive unfolding of proteins, thereby affecting their native structure and hindering subsequent amyloid fibril formation [22].

### 3.9 Analysis of correlations

**Fig. 8:**
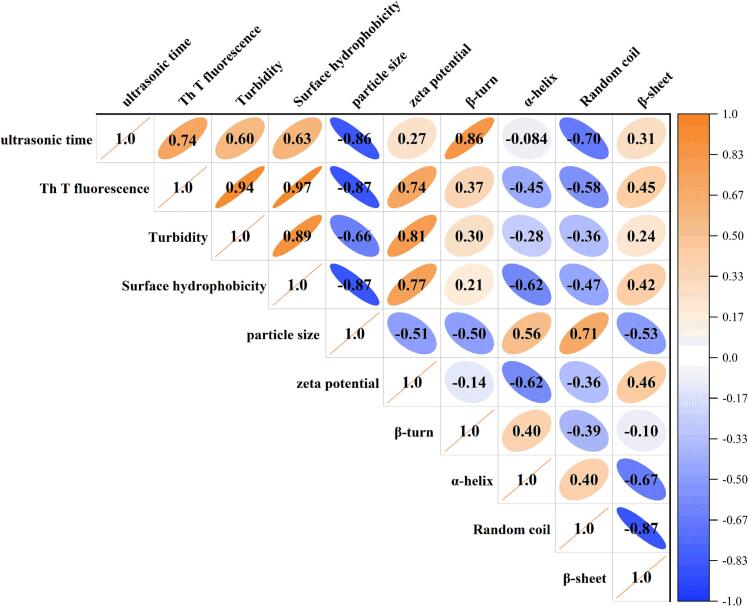
Statistical heat map of correlation analysis among different variables performed by Pearson's correlation analysis. Positive relationships are in orange, and negative relationships are in blue. (∗indicated a significant positive or negative correlation between two invariants, *P* < 0.05). (For interpretation of the references to color in this figure legend, the reader is referred to the Web version of this article.)

To investigate the effect of ultrasonic time on the fibrillation of OPI, the relationships between protein fibril structure and functionality were systematically analyzed using Pearson correlation coefficients. As shown in Fig. 8, ultrasonic time exhibited strong positive correlations with ThT fluorescence intensity, turbidity, surface hydrophobicity, zeta potential, and β‑sheet content. These trends are consistent with the results described above, confirming that ultrasonication-assisted significantly promotes the formation of β‑sheet structures. This observation can be explained by a hypothesis that, after ultrasonic pretreatment, more immature fibrils assemble and integrate into mature fibrils [50]. Moreover, β‑sheet content was positively correlated with ultrasonic time, ThT fluorescence intensity, turbidity, surface hydrophobicity, and zeta potential, whereas it was negatively correlated with particle size, α‑helix, β‑turn, and random coil structures. Similar observations have been reported in the ultrasonication‑assisted, acid‑heat induced self‑assembly and fibrillation of rice protein, where ultrasonic pretreatment accelerated protein hydrolysis and the self‑assembly process [73].

## 4 Conclusions

In this study, OPI was successfully converted into okara protein amyloid fibrils (OPF) through acid-heat (pH 2.0, 90 °C), demonstrating its capacity for structural rearrangement and self-assembly into β-sheet-rich fibrillar architecture. Moderate ultrasonic pretreatment enhances intermolecular interactions by promoting partial unfolding of the protein and exposing additional hydrophobic regions, thereby facilitating fibrillar formation. However, direct identification of the specific crosslinking mechanisms would require further characterization using techniques such as mass spectrometry. Similar findings have been reported in non-food protein systems, suggesting broader research implications for ultrasound-induced conformational regulation. Collectively, this study elucidates the mechanism by which ultrasonic pretreatment modulates the fibrillation behavior of plant proteins, offering a promising strategy for developing functional fiber materials derived from plants. These materials hold potential applications in food structure engineering, active substance delivery, and biomaterials.

## CRediT authorship contribution statement

**Shuangshuang Wang:** Writing – review & editing, Writing – original draft, Visualization, Project administration. **Haokun Zhang:** Writing – original draft, Software, Methodology, Investigation, Formal analysis, Data curation. **Huilin Lv:** Visualization, Methodology, Data curation. **Zhenzhu Li:** Resources, Methodology. **Shanshan Tie:** Visualization. **Fang Zhao:** Resources. **Peifeng Li:** Visualization, Supervision, Project administration.

## Declaration of competing interest

The authors declare that they have no known competing financial interests or personal relationships that could have appeared to influence the work reported in this paper.
