# Supplementary material for: Ultrasonic-assisted production of okara protein isolate amyloid fibrils from plant-based by-products: Structural and morphological characteristics
Source: Ultrason Sonochem. 2026 Apr 16;129:107857. doi: 10.1016/j.ultsonch.2026.107857 (PMC13125158; doi:10.1016/j.ultsonch.2026.107857)
Supplement: Supplementary Data 1 [file mmc1.docx]

Supplementary material

**Ultrasonic-assisted production of okara protein isolate amyloid fibrils from plant-based by-products: Structural and morphological characteristics**

Shuangshuang Wang ^a,b,c, d*^, Haokun Zhang ^a,b,c^, Huilin Lv ^a,b,c^, Zhenzhu Li ^a,b^, Shanshan Tie ^e^,

Fang Zhao ^f^, Peifeng Li ^a,b,c, *^

*^a^ College of Food and Bioengineering,* *Zhengzhou University of Light Industry,* *Zhengzhou, 450001, China*

*^b^ Key Laboratory of Cold Chain Food Processing and Safety Control, Ministry of Education, Zhengzhou University of Light Industry, Zhengzhou, 450001, China*

*^c^ Institute of Life and Health, Zhengzhou University of Light Industry, Zhengzhou, 450001, China*

*^d^ National & Local Joint Engineering Research Center of Cereal-Based Foods (Henan), Zhengzhou 450001, China*

*^e^ College of Food and Bioengineering, Henan Engineering Research Center of Food Microbiology, Henan University of Science and Technology, Luoyang 471023, China*

*^f^ Henan Institute of Product Quality Inspection Technology, Zhengzhou 450001, China*

^*^ Corresponding authors:

Shuangshuang Wang, Peifeng Li

Tel: + 86-371-86608670

E-mail: [wangsh052@zzuli.edu.cn](mailto:wangsh052@zzuli.edu.cn ) (S. Wang), [peifengli@zzuli.edu.cn](mailto:peifengli@zzuli.edu.cn) (P. Li)

**Table S1.** The effect of different ultrasonic treatment times on the diameter of OPI and OPF

| Samples | Diameter (nm) | Length (nm) |
| --- | --- | --- |
| OPI | 6.21±1.46 | 7.23±1.37 |
| OPF_0_ | 6.07±1.63 | 13.10±4.93 |
| OPF_5_ | 3.91±0.75 | 13.90±7.99 |
| OPF_10_ | 5.09±1.1 | 16.75±8.41 |
| OPF_15_ | 3.29±1.09 | 31.58±8.45 |
| OPF_20_ | 5.29±1.18 | 23.65±2.60 |
| OPF_25_ | 5.51±1.45 | 24.65±18.19 |
